# Risk Factors Associated With Quality of Life in Patients With Hepatitis B Virus Related Cirrhosis

**DOI:** 10.3389/fpsyg.2021.770415

**Published:** 2022-01-06

**Authors:** Qi Zhang, Chunxiu Zhong, Shaohang Cai, Tao Yu, Xuwen Xu, Junhua Yin

**Affiliations:** ^1^Department of Liver Disease Center, Shenzhen Hospital of Southern Medical University, Shenzhen, China; ^2^Department of Infectious Diseases, Nanfang Hospital, Southern Medical University, Guangzhou, China

**Keywords:** liver cirrhosis, mental disorder, antiviral treatment, health-related quality of life, chronic hepatitis B

## Abstract

**Aim:** To evaluate health-related quality of life (HRQoL) of chronic hepatitis B (CHB) and hepatitis B virus (HBV) related cirrhosis patients and analyzed specific differences in all dimensions of HRQoL.

**Methods:** A total of 349 patients met selection criteria were enrolled. The 36-Item Short-Form Health Survey was adopted.

**Results:** Results showed that the physiological HRQoL of the cirrhotic group was significantly lower than that of the non-cirrhotic group (*P* = 0.003), the psychological HRQoL was also lower (*P* = 0.006). HRQoL was significantly negatively correlated with liver stiffness (*P* = 0.001). We further evaluated the risk factors associated with poor HRQoL in HBV-related cirrhosis patients. Results showed that positive HBV DNA viral load (OR = 6.296, *P* = 0.041) and HCC family history (OR = 36.211, *P* = 0.001) were independent factors associated with HRQoL in HBV-related cirrhosis. For better risk stratification of patients, multivariable analyses were conducted to explore the independent factors that affected specific physiological and psychological HRQoL. In specific physiological HRQoL, results show that marital status (OR = 9.971, *P* = 0.034), positive HBV DNA viral load (OR = 6.202, *P* = 0.042) and antiviral drugs (OR = 0.45, *P* = 0.031) were independent factors associated with physiological HRQoL in cirrhosis patients. In psychological HRQoL, only HCC family history was independent risk factors associated with psychological HRQoL (OR = 42.684, *P* = 0.002).

**Conclusion:** We found that the impaired HRQoL dimensions of HBV related cirrhosis patients differ between the various subpopulations. According to our results, risk stratification, medical decision making and personalizing interventions could be made.

## Introduction

Hepatitis B virus (HBV) infection is a serious public health burden worldwide (Sarin et al., 2016). Epidemiological research has shown that approximately 240 million patients have a diagnosed chronic HBV infection (Lok and McMahon, 2009; Sarin et al., 2016). Among them, the Asia-Pacific region accounts for three-quarters of the global population (Sarin et al., 2016). Anti-HBV treatment is the most fundamental and important treatment for chronic hepatitis B (CHB) (European Association for the Study of the Liver [EASL], 2017). Currently, drugs that are licensed for anti-HBV treatment include interferon and nucleoside analogs (NAs). Although NAs can prevent the progression of hepatic impairment in CHB patients by inhibiting HBV replication, they cannot directly clear the cccDNA of HBV. Most patients with CHB who are treated with NAs need to receive therapy for a very long time.

For patients with HBV related liver cirrhosis, the anti-HBV treatments with NAs need to last a lifetime (Terrault et al., 2016; European Association for the Study of the Liver [EASL], 2017). Long-term use of NAs may cause to a decline in overall health resulting in a decline of health-related quality of life (HRQoL) and adverse effects in all aspects of social activities. International clinical guidelines have suggested that the goal of therapy for CHB is to improve the HRQoL (Sarin et al., 2016; European Association for the Study of the Liver [EASL], 2017). HRQoL is a multidimensional concept that includes physical, mental, emotional, and social functions (Guyatt et al., 1993; Sitlinger and Zafar, 2018). Previous studies have shown that CHB patients have lower quality of life than healthy people (Modabbernia et al., 2013; Zhuang et al., 2014). However, there have been many contradictions in previous researches. Moreover, it is not yet known HRQoL in HBV related cirrhosis patients and which dimensions are responsible for the decrease in HRQoL. Exploring the specific dimensions that cause a decline in the HRQoL of HBV-related cirrhosis patients could help to accurately intervene and improve their HRQoL.

Therefore, this study aims to evaluate the HRQoL of HBV-related cirrhosis patients and the related independent factors. In this study, we analyzed various sociodemographic factors and the specific differences in all dimensions of HRQoL and provide evidence-based medical evidence for targeted interventions.

## Subjects and Methods

### Subjects

We analyzed the HRQoL level of a group of HBV-related cirrhosis patients with regular follow-up in our center from 2015.01 to 2016.05. We defined the diagnosis of chronic HBV infection as serum hepatitis B surface antigen (HBsAg)-positive for at least 6 months and accompanied by an increase of serum ALT level (European Association for the Study of the Liver [EASL], 2012). The diagnosis of liver cirrhosis is based on imaging evidence or pathological evidence. Patients were excluded if they had hepatocellular carcinoma (HCC); co-infections with hepatitis C virus, hepatitis D virus, or HIV; any medical evidences of autoimmune hepatitis; heavy alcohol abuse; were pregnant; or were previously treated with interferon. The Institutional Review Board of Nanfang Hospital approved the study. Written informed consent were obtained from all patients enrolled. Socio-demographic information, such as gender, age, marital status, education level, and smoking and alcohol consumption history were collected for all participating subjects. The definitions of education levels were as follows: primary, less than 6 years of formal school education, secondary, 6–12 years of formal school education, and tertiary, university and postgraduate studies. All patients’ clinical data, including HBV DNA, ALT levels, and liver stiffness values were detected and recorded at the time of enrollment.

### Questionnaires

The 36-Item Short-Form Health Survey (SF-36) is a brief self-administered HRQoL instrument commonly used in various disease populations (McPherson and Martin, 2013; Arian et al., 2019). It includes eight items: physical functioning (PF), role limitations due to physical problems (RP), bodily pain (BP), general health (GH), vitality (VT), social functioning (SF), role limitations due to emotional problems (RE), and mental health (MH). In addition, there are two summary measures: the physiological (PF, RP BP, and GH) and psychological HRQoL (VT, SF, RE, and MH). These items with higher scores mean better health conditions. The Chinese versions of the SF-36 questionnaire were available and provided by the developer. The tool’s validity and screening ability have been documented in various samples in China.

### Statistical Analysis

Continuous variables were expressed as mean and standard deviation, and categorical variables were expressed as percentages. The Chi-square test and Student’s *t*-test were applied as appropriate, to determine statistically significant differences. Univariate and multivariate analyses were used to find factors associated with QoL. The statistical significance of all tests was set as *p* < 0.05 by two-tailed tests. Data analyses and quality control procedures were performed using SPSS for Windows, version 13.0 (SPSS Inc., Chicago, United States).

## Results

### Demographic Data in Chronic Hepatitis B Patients With or Without Cirrhosis

A total of 349 patients completed the SF-36 questionnaire. A total of 88 patients were diagnosed with cirrhosis while 261 without cirrhosis. The weight of patients in the cirrhosis group was heavier than that in the non-cirrhosis group (*P* = 0.02). The characteristics of patients are shown in Table 1.

**TABLE 1:** Demographics data in patents enrolled.

Characteristics	Chronic hepatitis B patients		*P*-value
	With cirrhosis (*n* = 88)	Without cirrhosis (*n* = 261)	
Sex			0.900
Male	76 (86.4%)	224 (85.8%)	
Female	12 (13.6%)	37 (14.2%)	
Age, years	38.84 ± 10.09	37.76 ± 9.64	0.345
Height, cm	167.52 ± 7.38	168.00 ± 6.16	0.519
Weight, Kg	65.87 ± 12.46	62.95 ± 9.98	0.020
Education level			<0.001
Primary	16 (18.2%)	13 (5.0%)	
Secondary	48 (54.5%)	114 (43.7%)	
Tertiary	24 (27.3%)	134 (51.3%)	
Marital status			0.002
Unmarried	4 (4.5%)	53 (20.3%)	
Married	81 (92.1%)	202 (77.4%)	
Divorced	3 (3.4%)	6 (2.3%)	

### Health-Related Quality of Life in Chronic Hepatitis B Patients With or Without Cirrhosis

We compared HRQoL in cirrhosis group and non-cirrhosis group. Results showed that HRQoL in patients with cirrhosis were significantly lower than in patients without cirrhosis, specific in dimension of PF, VT and SF (Supplementary Figure 1A). Therefore, we analyzed the difference between total physiological HRQoL and total psychological HRQoL between the two groups (Supplementary Figure 1B). The physiological HRQoL of the cirrhotic group was significantly lower than that of the non-cirrhotic group (*P* = 0.003), and the psychological HRQoL was also lower (*P* = 0.006). In order to further analyze the relationship between liver cirrhosis and HRQoL, we analyzed the relationship between liver stiffness and HRQoL. The results showed that HRQoL was significantly negatively correlated with liver stiffness (*P* = 0.001, Supplementary Figure 1C).

We further conducted a multivariable analysis to explore the independent factors that affected patients with CHB. Results show that cirrhosis were independent factors that affected the HRQoL (OR = 0.478, *P* = 0.010), as shown in Supplementary Table 1.

### Factor of Health-Related Quality of Life in Patients With Hepatitis B Virus-Related Cirrhosis

Based on the mean HRQoL, patients were divided into two groups: those with better HRQoL and those with poor HRQoL. Results showed that patients with HCC family history had significantly lower HRQoL (*P* = 0.003). As shown in Table 2. Hence, we evaluated the risk factors associated with poor HRQoL in HBV-related cirrhosis patients (Supplementary Table 2). Results showed that positive HBV DNA viral load (OR = 6.296, *P* = 0.041) and HCC family history (OR = 36.211, *P* = 0.001) were independent factors associated with HRQoL in HBV-related cirrhosis.

**TABLE 2:** Factors of health-related quality of life in cirrhosis patients.

Characteristics	Health related quality of life		*P*-value
	≥75 (*n* = 49)	<75 (*n* = 39)	
**Sex**			0.293
Male	44 (89.8%)	32 (82.1%)	
Female	5 (10.2%)	7 (17.9%)	
**Age, years**	38.25 ± 9.52	39.56 ± 11.06	0.553
**Height, cm**	168.24 ± 7.54	166.21 ± 6.89	0.198
**Weight, Kg**	66.04 ± 10.29	65.26 ± 15.06	0.777
**Education level**			0.343
Primary	7 (14.3%)	9 (23.1%)	
Secondary	26 (53.1%)	22 (56.4%)	
Tertiary	16 (32.7%)	8 (20.5%)	
**Marital status**			0.545
Unmarried	3 (6.1%)	1(2.6%)	
Married	45 (91.8%)	36 (92.3%)	
Divorced	1 (2.0%)	2 (5.1%)	
**Exercise, Y/N**	36/13	30/9	0.710
**Smoking, Y/N**	17/32	14/25	0.907
**Alcohol consumption, Y/N**	19/30	16/23	0.830
**Family history, Y/N**	2/47	10/29	0.003
**HBV DNA, P/N**	5/44	7/32	0.293
**ALT level, U/L**	32.67 (±16.14	33.85 (±15.32	0.730
Treatment duration, years	5.39 ± 2.95	5.62 ± 3.68	0.748
**Antiviral treatment**			0.229
Lamivudine	2 (4.1%)	2 (5.1%)	
Adefovir	2 (4.1%)	3 (7.7%)	
Telbivudine	0 (0%)	3 (7.7%)	
Entecavir	38 (77.6%)	25 (64.1%)	
Tenofovir	7 (14.3)	6 (15.4%)	

### Impaired Dimensions of Health-Related Quality of Life Caused by Hepatitis B Virus DNA and Family History

We next determined which dimension of HRQoL were decreased by HBV DNA and family history. Results showed that patients with positive serum HBV DNA viral load had lower RP than the other patients (Supplementary Figure 2A). In addition, for cirrhosis patients with HCC family history, all the psychological dimensions of HRQoL including VT, SF, RE and MH were lower than the other patients (Supplementary Figure 2B).

### Independent Factors Associated With Physiological and Psychological Health-Related Quality of Life

For better risk stratification of patients, univariable and multivariable analyses were conducted to explore the independent factors that affected specific physiological and psychological HRQoL. In specific physiological HRQoL (Table 3), results show that marital status (OR = 9.971, *P* = 0.034), positive HBV DNA viral load (OR = 6.202, *P* = 0.042) and antiviral drugs (OR = 0.45, *P* = 0.031) were independent factors associated with physiological HRQoL in cirrhosis patients. In psychological HRQoL (Table 4), only HCC family history was independent risk factors associated with psychological HRQoL (OR = 42.684, *P* = 0.002).

**TABLE 3:** Multivariable analysis for physiological quality of life.

Variables	Univariate analysis			Multivariate analysis		
	OR	95% CI	P	OR	95% CI	P
Sex	1.556	0.453–5.337	0.482	0.349	0.044–2.763	0.319
Age	1.014	0.973–1.057	0.514	1.038	0.980–1.100	0.198
Height	0.964	0.908–1.023	0.230	0.932	0.830–1.046	0.232
Weight	0.988	0.954–1.024	0.508	0.966	0.910–1.025	0.247
Education level	0.668	0.353–1.264	0.215	0.447	0.184–1.089	0.076
Exercise	1.200	0.456–3.159	0.712	1.665	0.456–6.088	0.441
Marital status	4.475	0.716–27.981	0.109	9.971	1.184–83.959	0.034
ALT level	1.003	0.977–1.031	0.799	1.011	0.971–1.053	0.587
HBV DNA	2.343	0.650–8.445	0.193	6.202	1.068–36.014	0.042
Family history	1.556	0.453–5.337	0.482	3.999	0.778–20.567	0.097
Treatment duration	1.030	0.906–1.172	0.648	1.004	0.843–1.196	0.960
Antiviral drugs	0.603	0.352–1.032	0.065	0.450	0.218–0.930	0.031
Smoking	0.795	0.331–1.912	0.609	0.588	0.174–1.985	0.392
Alcohol consumption	0.811	0.345–1.908	0.631	0.588	0.174–1.985	0.392

**TABLE 4:** Multivariable analysis for psychological quality of life.

Variables	Univariate analysis			Multivariate analysis		
	OR	95% CI	P	OR	95% CI	P
Sex	1.556	0.453–5.337	0.482	0.892	0.115–6.930	0.913
Age	1.001	0.961–1.044	0.951	1.037	0.981–1.096	0.194
Height	0.970	0.915–1.029	0.318	0.953	0.854–1.064	0.393
Weight	1.002	0.969–1.037	0.901	1.014	0.961–1.069	0.615
Education level	0.537	0.279–1.035	0.063	0.415	0.164–1.049	0.063
Exercise	0.738	0.280–1.944	0.539	0.852	0.233–3.119	0.809
Marital status	1.719	0.579–5.107	0.329	1.982	0.523–7.502	0.314
ALT level	1.008	0.981–1.035	0.570	1.015	0.976–1.055	0.456
HBV DNA	1.556	0.453–5.337	0.482	2.923	0.564–15.161	0.201
Family history	15.125	1.857–123.162	0.011	42.684	3.914–465.539	0.002
Treatment duration	0.987	0.868–1.122	0.844	0.956	0.803–1.137	0.609
Antiviral drugs	1.113	0.694–1.783	0.657	0.888	0.484–1.629	0.701
Smoking	1.448	0.601–3.486	0.409	1.841	0.555–6.111	0.318
Alcohol consumption	1.186	0.505–2.787	0.696	0.389	0.111–1.362	0.140

## Discussion

Patients chronic infected with HBV suffer not only from impaired physical health but also mood disorders, which both affect their HRQoL (Modabbernia et al., 2013). The HRQoL of patients with CHB is obviously inferior to that of general population (Bao et al., 2007; Karaivazoglou et al., 2010). Although the impairment of HRQoL in CHB patients has been confirmed, the difference in the HRQoL dimensions of CHB patients in different subpopulations is unknown. In our study, we confirmed that the HRQoL of CHB patients was decreased. We further found that HRQoL in patients with cirrhosis were further decreased. We also found that the impaired HRQoL dimensions vary among different subpopulations of CHB patients.

According to the results of our study, the dimensions of decreased HRQoL in different populations of CHB patients are not the same. The eight dimensions of HRQoL are as follows: (1) PF: health condition hinders regular physiological activities; (2) RP: functional limitations caused by physiological health problems; (3) BP: the degree of pain and the impact of pain on daily activities; (4) GH: self-feeling of their own health status and disease prognosis; (5) VT: self-feeling of their own energy and fatigue level; (6) SF: the impact of physical and psychological problems on the quantity and quality of social activities; (7) RE: functional limitations of social roles caused by emotional problems; (8) MH: depression, behavioral or emotional disorder. In our study, we found that positive HBV DNA viral load patients are more likely to have a deceased RP. In addition, for cirrhosis patients with HCC family history, all the dimensions of psychological HRQoL including VT, SF RE and MH are decreased. Different measures can be taken for cirrhosis patients who are impaired in different dimensions of HRQoL. These measures include humanism, such as establishing a sincere relationship with friends and increasing social support; cognitive therapy, such as providing health knowledge education to help patients understand the disease; and behavioral therapy, such as learning how to accurately express emotions. In addition, it is also important to rebuild the social role of patients and increase some beneficial social activities.

In the management of HBV infection, it is very important to inform patients to keep adhering to their antiviral treatment plan in order to improve their HRQoL. In addition, according to the results of our study, more attention should be paid to positive HBV DNA viral load patients and positive HCC family history patients. Timely intervention should be provided to help solve physiological and psychological discomforts and improve HRQoL.

Cortesi et al. (2020) reported that decompensated cirrhosis and HCC patients reported higher problems in all physical domains (mobility, self-care and in performing usual activities evaluated by EQ-5D-3L), while only decompensated cirrhosis had more problems in the anxiety/depression domain. In our study, we found that the impaired HRQoL dimensions vary among different subpopulations of CHB patients. Specifically, we observed that HCC family history is an independent risk factors associated with psychological HRQoL in cirrhosis patient. According to guidelines for the prevention and treatment of chronic hepatitis B (Version 2019) recommended by Chinese Society of Infectious Diseases (Chinese Society of Infectious Diseases Chinese Medical Association, and Chinese Society of Hepatology, Chinese Medical Association, 2019). HCC family history should be listed as one of the indicators for initiating antiviral therapy. According to our study, we found that the HCC family history should also be listed as one of the indicators to initiate psychological evaluation and timely intervention.

There are some limitations of our study. Some social characteristics were not included in the statistics. Since we did not intervene the patient, we failed to analyze the changes in the quality of life of patients during the follow-up. For the analysis on the change of HRQoL of the patients during the follow-up after intervention requires additional studies to confirm. In addition, because all the patients were from one medical center, this study lacked a prospective multicenter cohort study to validate our results.

## Conclusion

In the study, we found that the HRQoL of cirrhosis patients was decreased. Moreover, we noticed that the impaired HRQoL dimensions of cirrhosis patients varied among different subpopulations. Hence, according our results, cirrhosis patients with high risk of poor HRQoL could be identified and risk stratification could be implemented.

## Data Availability Statement

The raw data supporting the conclusions of this article will be made available by the authors, without undue reservation.

## Ethics Statement

The studies involving human participants were reviewed and approved by the Nanfang Hospital, Southern Medical University. The patients/participants provided their written informed consent to participate in this study.

## Author Contributions

JY and XX conceived and designed the study. SC and QZ analyzed and interpreted the data, drafted, and finalized the manuscript. CZ, QZ, XX, and TY participated in the recruitment of patients, data collection, and critical revision of the article. All authors critically reviewed the final version of the manuscript.

## Conflict of Interest

The authors declare that the research was conducted in the absence of any commercial or financial relationships that could be construed as a potential conflict of interest.

## Publisher’s Note

All claims expressed in this article are solely those of the authors and do not necessarily represent those of their affiliated organizations, or those of the publisher, the editors and the reviewers. Any product that may be evaluated in this article, or claim that may be made by its manufacturer, is not guaranteed or endorsed by the publisher.

## Supplementary Material

Supplementary Figure 1 The difference of HRQoL between CHB patients and HBV-related cirrhosis patients. **(A)** PF score in cirrhosis was significantly lower than in CHB patients (89.09 ± 13.33 vs. 93.12 ± 10.63, *P* = 0.002), similar in VT score (64.69 ± 18.89 vs. 70.53 ± 16.96, *P* = 0.004), SF score (73.69 ± 21.89 vs. 79.41 ± 19.15, *P* = 0.013) and in total score (73.07 ± 14.19 vs. 77.44 ± 13.61, *P* = 0.006). **(B)** Physiological HRQoL in cirrhosis patients was 79.11 ± 12.15, significantly lower than in CHB patients of 83.49 ± 10.77 (*P* = 0.003), similar in psychological HRQoL (72.49 ± 13.68 vs. 77.59 ± 12.88, *P* = 0.006). **(C)** Liver stiffness was significantly negative correlated with HRQoL (*R* = -0.21, *P* = 0.001).

Supplementary Figure 2 Impaired dimensions of HRQoL caused by HBV DNA and family history. **(A)** RP score in positive HBV DNA viral load patients was 66.67 ± 32.57, significantly lower than negative HBV DNA viral load patients with 86.51 ± 19.34 (*P* = 0.004). **(B)** VT score in HCC family history was 53.75 ± 17.47, lower than the others with 68.28 ± 18.52 (*P* = 0.013), similar in SF score (61.11 ± 18.65 vs. 77.05 ± 21.57, *P* = 0.018), RE score (69.44 ± 26.43 vs. 85.09 ± 23.97, *P* = 0.041), and MH score (57.33 ± 11.97 vs. 67.16 ± 17.16, *P* = 0.023).

## Abbreviations

HRQoL: health-related quality of life
CHB: chronic hepatitis B
HBV: Hepatitis B virus
NAs: nucleoside analogs
HBsAg: hepatitis B surface antigen
SF-36: 36-Item Short-Form Health Survey
PF: physical functioning
RP: role limitations due to physical problems
BP: bodily pain
GH: general health
VT: vitality
SF: social functioning
RE: role limitations due to emotional problems
MH: mental health.
