# Supplementary material for: Risk Factors Associated With Quality of Life in Patients With Hepatitis B Virus Related Cirrhosis
Source: Front Psychol. 2022 Jan 6;12:770415. doi: 10.3389/fpsyg.2021.770415 (PMC8770820; doi:10.3389/fpsyg.2021.770415)
Supplement: Supplementary file 1 [file Table_1.DOCX]

**Supplementary Table 1.** **Multivariable analysis for quality of life in CHB patients**

| **Variables** | **Univariate analysis** | | | **Multivariate analysis** | | |
| --- | --- | --- | --- | --- | --- | --- |
|  | **OR** | **95% CI** | **P** | **OR** | **95% CI** | **P** |
| Sex | 2.166 | 1.168-4.017 | 0.014 | 1.780 | 0.737-4.298 | 0.200 |
| Age | 1.011 | 0.988-1.035 | 0.342 | 1.009 | 0.984-1.035 | 0.476 |
| Height | 0.963 | 0.929-0.998 | 0.040 | 1.002 | 0.949-1.058 | 0.940 |
| Weight | 0.985 | 0.962-1.008 | 0.188 | 0.992 | 0.963-1.022 | 0.603 |
| Education level | 0.592 | 0.412-0.851 | 0.005 | 0.742 | 0.484-1.137 | 0.171 |
| Exercise | 0.505 | 0.292-0.871 | 0.014 | 0.595 | 0.325-1.089 | 0.092 |
| Marital status | 1.212 | 0.773-1.901 | 0.402 | 1.091 | 0.658-1.810 | 0.735 |
| ALT level | 0.997 | 0.986-1.009 | 0.658 | 1.000 | 0.987-1.014 | 0.944 |
| HBV DNA | 0.857 | 0.433-1.696 | 0.658 | 0.823 | 0.367-1.849 | 0.638 |
| Family history | 1.585 | 0.858-2.926 | 0.141 | 1.770 | 0.912-3.437 | 0.092 |
| Treatment duration | 0.946 | 0.891-1.004 | 0.069 | 0.957 | 0.889-1.031 | 0.250 |
| Antiviral drugs | 0.969 | 0.761-1.236 | 0.802 | 0.867 | 0.654-1.150 | 0.323 |
| Smoking | 1.176 | 0.708-1.952 | 0.531 | 1.180 | 0.641-2.171 | 0.596 |
| Alcohol consumption | 1.052 | 0.649-1.706 | 0.836 | 0.825 | 0.461-1.475 | 0.516 |
| Liver cirrhosis | 0.417 | 0.251-0.691 | 0.001 | 0.478 | 0.274-0.836 | **0.010** |
