# Supplementary material for: Risk Factors Associated With Quality of Life in Patients With Hepatitis B Virus Related Cirrhosis
Source: Front Psychol. 2022 Jan 6;12:770415. doi: 10.3389/fpsyg.2021.770415 (PMC8770820; doi:10.3389/fpsyg.2021.770415)
Supplement: Supplementary file 2 [file Table_2.DOCX]

**Supplementary Table 2.** **Multivariable analysis for quality of life in cirrhosis patients**

| **Variables** | **Univariate analysis** | | | **Multivariate analysis** | | |
| --- | --- | --- | --- | --- | --- | --- |
|  | **OR** | **95% CI** | **P** | **OR** | **95% CI** | **P** |
| Sex | 1.925 | 0.560-6.617 | 0.299 | 0.915 | 0.106-7.904 | 0.935 |
| Age | 1.013 | 0.971-1.056 | 0.549 | 1.057 | 0.995-1.123 | 0.074 |
| Height | 0.961 | 0.905-1.021 | 0.199 | 0.922 | 0.818-1.039 | 0.183 |
| Weight | 0.995 | 0.961-1.030 | 0.774 | 1.003 | 0.950-1.058 | 0.921 |
| Education level | 0.621 | 0.325-1.185 | 0.148 | 0.400 | 0.152-1.054 | 0.064 |
| Exercise | 1.204 | 0.453-3.202 | 0.710 | 1.826 | 0.469-7.118 | 0.385 |
| Marital status | 1.750 | 0.599-5.119 | 0.306 | 2.572 | 0.585-11.309 | 0.211 |
| ALT level | 1.005 | 0.978-1.032 | 0.727 | 1.022 | 0.980-1.065 | 0.315 |
| HBV DNA | 1.925 | 0.560-6.617 | 0.299 | 6.296 | 1.079-36.745 | **0.041** |
| Family history | 8.103 | 1.657-39.625 | 0.010 | 36.211 | 4.236-309.570 | **0.001** |
| Treatment duration | 1.022 | 0.898-1.162 | 0.745 | 1.013 | 0.846-1.212 | 0.892 |
| Antiviral drugs | 0.809 | 0.503-1.300 | 0.381 | 0.588 | 0.308-1.124 | 0.108 |
| Smoking | 1.054 | 0.437-2.541 | 0.907 | 1.339 | 0.399-4.498 | 0.637 |
| Alcohol consumption | 1.098 | 0.465-2.592 | 0.830 | 0.432 | 0.120-1.556 | 0.199 |
