# Supplementary figures and images for: Risk Factors Associated With Quality of Life in Patients With Hepatitis B Virus Related Cirrhosis
Source: Front Psychol. 2022 Jan 6;12:770415. doi: 10.3389/fpsyg.2021.770415 (PMC8770820; doi:10.3389/fpsyg.2021.770415)

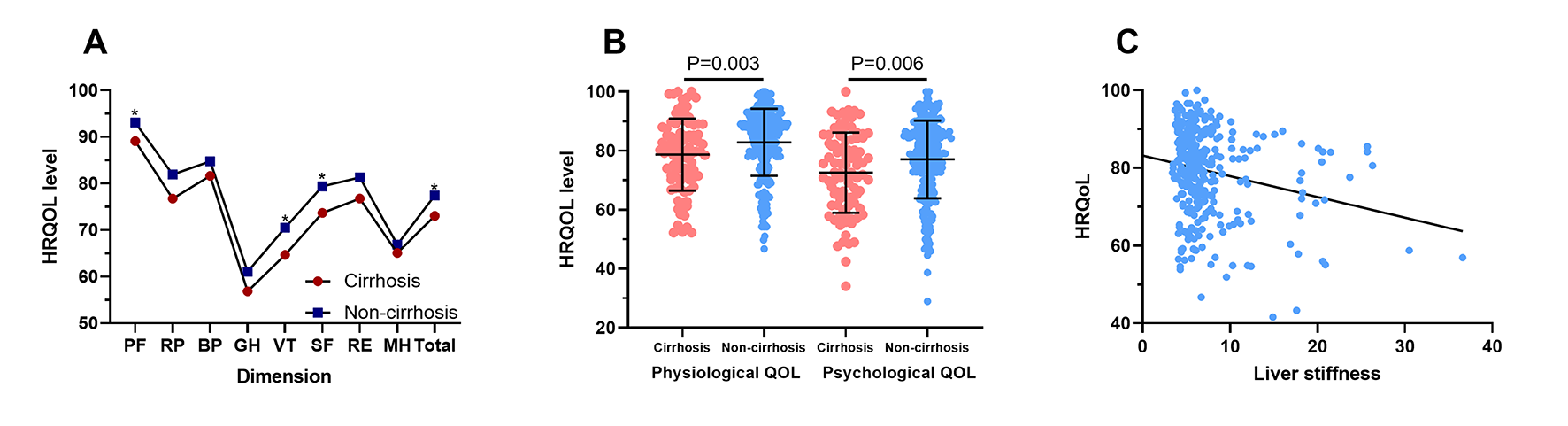

Supplement: Supplementary Figure 1 — The difference of HRQoL between CHB patients and HBV-related cirrhosis patients. (A) PF score in cirrhosis was significantly lower than in CHB patients (89.09 ± 13.33 vs. 93.12 ± 10.63, P = 0.002), similar in VT score (64.69 ± 18.89 vs. 70.53 ± 16.96, P = 0.004), SF score (73.69 ± 21.89 vs. 79.41 ± 19.15, P = 0.013) and in total score (73.07 ± 14.19 vs. 77.44 ± 13.61, P = 0.006). (B) Physiological HRQoL in cirrhosis patients was 79.11 ± 12.15, significantly lower than in CHB patients of 83.49 ± 10.77 (P = 0.003), similar in psychological HRQoL (72.49 ± 13.68 vs. 77.59 ± 12.88, P = 0.006). (C) Liver stiffness was significantly negative correlated with HRQoL (R = -0.21, P = 0.001). [file Image_1.TIF]

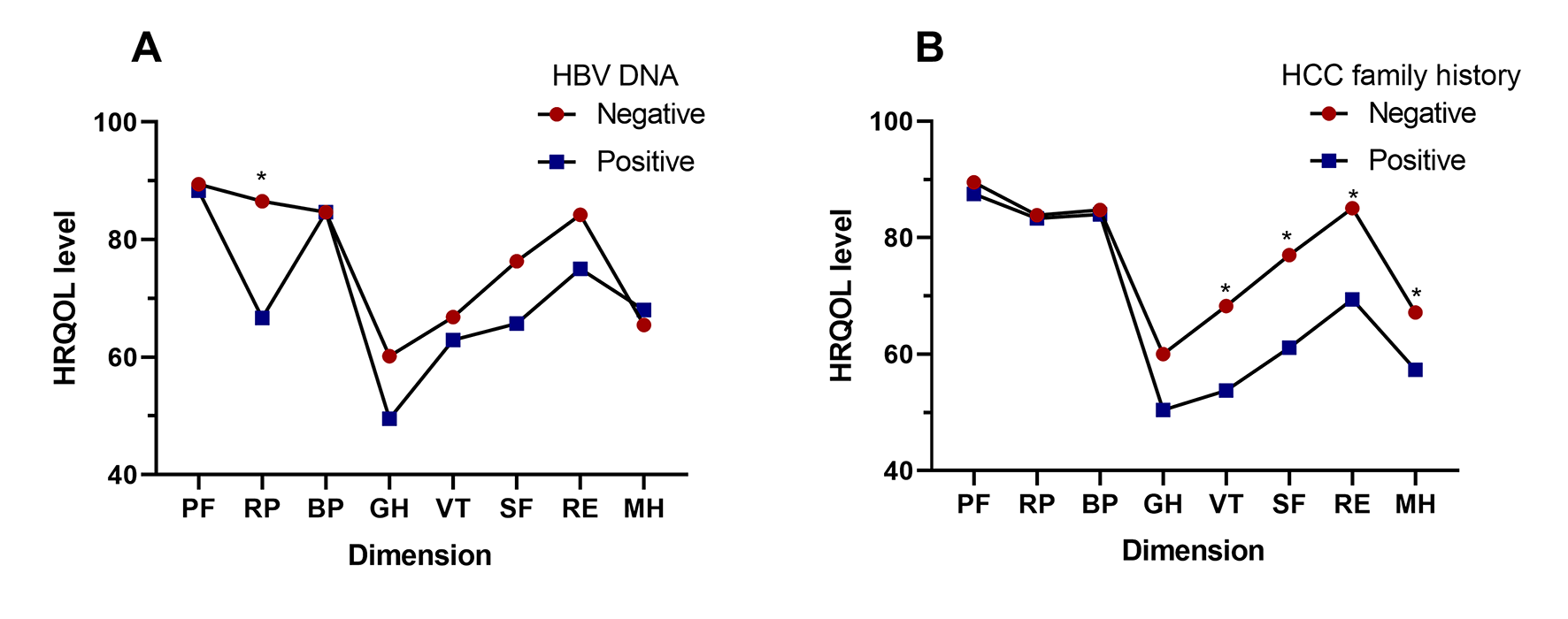

Supplement: Supplementary Figure 2 — Impaired dimensions of HRQoL caused by HBV DNA and family history. (A) RP score in positive HBV DNA viral load patients was 66.67 ± 32.57, significantly lower than negative HBV DNA viral load patients with 86.51 ± 19.34 (P = 0.004). (B) VT score in HCC family history was 53.75 ± 17.47, lower than the others with 68.28 ± 18.52 (P = 0.013), similar in SF score (61.11 ± 18.65 vs. 77.05 ± 21.57, P = 0.018), RE score (69.44 ± 26.43 vs. 85.09 ± 23.97, P = 0.041), and MH score (57.33 ± 11.97 vs. 67.16 ± 17.16, P = 0.023). [file Image_2.TIF]
